# The effectiveness of ERCP in managing preoperative complications of choledochal cysts in children and its role in facilitating early surgical intervention

**DOI:** 10.3389/fped.2025.1523753

**Published:** 2025-01-24

**Authors:** Tian Zhang, Wenjie Wu, Yijun Shu, Hao Weng, Mingzhe Weng, Ying Zhou, Xuefeng Wang

**Affiliations:** ^1^ Department of Pediatric Surgery, Xinhua Hospital Affiliated to Shanghai Jiao Tong University School of Medicine, Shanghai, China; ^2^ Department of General Surgery, Xinhua Hospital Affiliated to Shanghai Jiao Tong University School of Medicine, Shanghai, China

**Keywords:** children, ERCP, choledochal cyst, complication, surgery

## Abstract

**Introduction:**

Currently, there is no established guideline for the application of ERCP in children with choledochal cyst. This study aimed to investigate the safety and effectiveness of ERCP in managing preoperative complications of choledochal cyst in children, as well as the timing for definitive surgery following ERCP.

**Methods:**

We conducted a retrospective review of medical records for 68 pediatric patients who presented with complications of choledochal cyst, including pancreatitis and biliary obstruction combined with cholangitis. All patients underwent ERCP treatment followed by definitive surgery. The primary outcomes assessed included treatment efficacy, post-ERCP complication, and the impact of ERCP on definitive surgical procedures.

**Results:**

Among the 68 patients studied, 41 presented with pancreatitis, while the remaining patients had biliary obstruction and cholangitis. Sixty-five patients successfully completed their treatments, with 64 experiencing alleviation of symptoms. Significant improvements were observed in serum amylase levels and liver function tests following ERCP. Post-ERCP complications occurred in three cases, including one case of pancreatitis and two cases of infection. The median interval between ERCP and surgery was 11 days. There was no significant difference in primary outcomes, such as surgical duration, rate of minimally invasive surgery, conversion to open surgery, intraoperative bleeding volume, intraoperative blood transfusion, postoperative complications, or average length of hospital stay, between the early surgery group (≤2 weeks) and the late surgery group (>2 weeks).

**Conclusions:**

ERCP was proved to be a safe and effective intervention for alleviating preoperative complications in pediatric patients with choledochal cyst. Early definitive surgery following ERCP did not significantly impact the perioperative outcomes of pediatric patients.

## Introduction

Choledochal cyst is a common congenital anomaly in children (1). It is more prevalent in the Asian population, with an incidence rate of approximately one in ten thousand. It typically presents as dilation of the common bile duct, with or without concurrent intrahepatic bile duct dilation. The standard curative approach involves cyst excision along with hepaticojejunostomy using Roux-en-Y anastomosis (2). Currently, it is believed that the pathogenesis of choledochal cyst is based on the pancreaticobiliary maljunction (PBM) (3). The convergence of the bile and pancreatic ducts outside the wall of the duodenum leads to an excessively long common channel. Dysregulation of the Oddi sphincter disrupts the fluid dynamics stability of bile and pancreatic secretions, causing widespread pancreatic fluid reflux into the common bile duct, further leading to chronic inflammation of the bile ducts and ultimately increasing the risk of bile duct cancer (4).

Minimally invasive surgery has been widely used in the treatment of children with choledochal cyst. Children with choledochal cyst may experience various complications before surgery, such as bile duct obstruction, cholangitis, bile duct perforation, and pancreatitis (5). Once these complications occur, they often lead to the postponement of definitive surgery or necessitate emergency open surgery. Additionally, local inflammation and other factors increase the difficulty of minimally invasive surgery (6, 7). Therefore, there is not yet a fully unified consensus on the timing of definitive surgery for children with choledochal cyst who present with preoperative complications.

Endoscopic retrograde cholangiopancreatography (ERCP) is an important diagnostic and therapeutic method for biliary and pancreatic diseases, and its minimally invasive nature and effectiveness have led to its rapid development (8, 9). ERCP procedures are often challenging in pediatric patients, and specific guidelines for their use have not yet been established (10). However, ERCP has gradually been widely used in the diagnosis and treatment of pediatric hepato-biliary-pancreatic diseases. For the diagnosis of PBM (pancreaticobiliary maljunction) in children with choledochal cyst, ERCP is more accurate than ultrasound and magnetic resonance cholangiopancreatography (MRCP) (11, 12). However, due to the requirement for general anesthesia in pediatric ERCP procedures, most doctors do not recommend performing diagnostic ERCP alone in children. This study aims to explore the safety and effectiveness of ERCP in treating preoperative complications of choledochal cyst in children and the timing of definitive surgery after ERCP.

## Materials and methods

### Patients and data collection

This study retrospectively enrolled pediatric patients who underwent choledochal cyst surgery at Xinhua Hospital from Jan 2016 to Dec 2022. The study has been authorized by the Ethics Committee of Xinhua Hospital. Inclusion criteria were as follows: (1) Patients diagnosed with choledochal cyst based on imaging examinations including ultrasound, MRCP, etc., and underwent preoperative ERCP and definitive surgical treatment at our center; (2) Patients with complications related to choledochal cyst including (1) biliary obstruction combined with cholangitis, (2) pancreatitis.

Exclusion criteria included: (1) Patients with Todani type 5 choledochal cyst; (2) Patients with PBM but no extrahepatic bile duct dilation; (3) Patients with existing biliary perforations.; (4) Patients who, despite undergoing ERCP treatment, did not receive radical surgical treatment. According to the inclusion and exclusion criteria, a total of 68 patients were included in the study. Demographic characteristics, clinical presentations, laboratory data, and imaging findings were gathered and compare.

### Surgical strategy

After admission, pediatric surgeons selected patients who required ERCP treatment based on the child's symptoms, physical signs, laboratory tests, and imaging characteristics. During the surgical procedure, endotracheal intubation and a combination of intravenous and inhalation anesthesia were employed.

### ERCP procedure

Patients were placed in the prone position with the external genitalia and thyroid gland covered. In the study, all children were examined using a duodenoscope (Olympus, JF260V). After insertion, the main papilla and accessory papilla of the duodenum were observed first. The main papilla was cannulated to observe the dilation of the common bile duct, the presence of stones in the pancreaticobiliary duct, and to check for the presence of PBM and congenital pancreatic duct structural abnormalities. After pancreaticobiliary duct imaging, endoscopic sphincterotomy (EST) was performed and stones was extracted using a sphincterotome, retrieval balloon, or stone retrieval basket. Antibiotics were routinely administered after ERCP, and serum amylase levels were checked at 6 and 24 h postoperatively. If the child showed signs of postoperative pancreatitis, serum amylase levels were further monitored at 48 or 72 h postoperatively, and enzyme inhibitors were administered.

### Post-ERCP complications

Monitoring post-ERCP pancreatitis (PEP), perforation, bleeding, and infection. Post-ERCP pancreatitis (PEP) was defined as a rise in serum amylase levels exceeding three times the upper limit of normal within 24–72 h post-ERCP, coupled with abdominal pain.

### Definitive surgery

The patients accepting definitive surgery all underwent choledochal cyst excision and hepaticojejunostomy with Roux-en-Y anastomosis. We divided all patients into an early surgery group and a late surgery group based on a time point of 2 weeks after ERCP to compare perioperative conditions and short-term outcomes.

### Statistical analysis

Continuous data were expressed as mean ± standard deviation, while categorical data were presented as rates. The comparison of continuous data was performed using the un-paired *T* test or paired *T*-test, while the comparison of categorical data was conducted using the chi-square test. Analysis was conducted using SPSS 22.0 software. *P* < 0.05 indicated statistical significance, and all *P* values were two-tailed.

## Results

### Demographics and characteristics of patients

This retrospective study included a total of 68 children, whose basic information is shown in Table 1. There were 24 boys and 44 girls. The average age was 47.4 ± 30.8 months, with an average weight of 15.1 ± 5.9 kg. There were 58 cases presenting with abdominal pain, 20 cases with vomiting, 16 cases with jaundice and 5 cases with fever. Additionally, there were 2 cases with pale stool. Among the 68 children, 27 presented with biliary obstruction combined with cholangitis, while 41 showed symptoms of pancreatitis. Among those with pancreatitis, 36 children also exhibited elevated γ-GT and liver enzyme levels, with or without elevated direct bilirubin levels. MRCP or computed tomography (CT) scan was routinely performed for children with choledochal cysts.

**Table 1:** Baseline characteristics of 68 patients.

Events	Values
Age (months)	47.4 ± 30.8
Gender (male/female)	24/44
Weight (Kg)	15.1 ± 5.9
Clinical manifestations	
Abdominal pain	58
Vomiting	20
Fever	5
Jaundice	16
Clay stool	2
Complications	
Pancreatitis	41
Biliary obstruction and cholecystitis	27

### ERCP interventions

**Figure 1:**
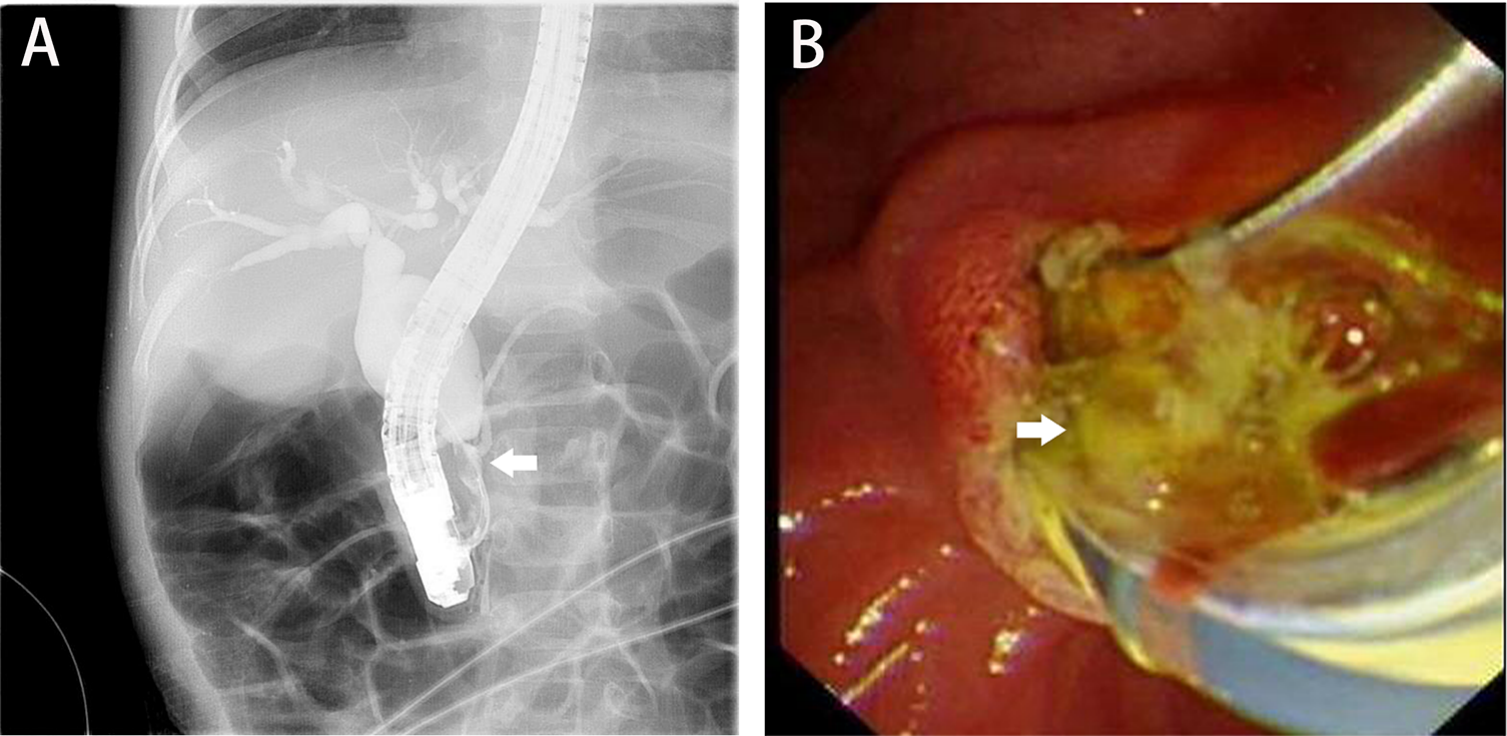
Intraoperative ERCP images. **(A)** The dilated common bile duct and a suspected filling defect in the common channel, with the white arrow indicating PBM; **(B)**, the white arrow points to the extracted protein plugs. ERCP, endoscopic retrograde cholangiopancreatography; PBM, pancreaticobiliary maljunction.

Among 68 pediatric patients, two cases failed to undergo cannulation, one case only completed cholangiography due to the inability of the guide wire to enter the common bile duct (Figure 1A), and the remaining 65 cases completed treatment. All 65 pediatric patients underwent EST. Four pediatric patients did not show obvious signs of stone formation on imaging and only underwent endoscopic nasobiliary drainage (ENBD). The remaining 61 pediatric patients, except for two cases where stone extraction was difficult and only ENBD was performed, all underwent stone extraction (Figure 1B). All the stones in the patients were protein plugs. The methods of stone extraction included the use of a sphincterotomy knife in 43 cases, stone retrieval basket in 15 cases and stone retrieval balloon in one case. In the process of stone extraction, dilation balloons were used as an adjunct in two pediatric cases. Postoperative drainage methods included 64 cases of ENBD and 1 case of endoscopic retrograde pancreatic drainage (ERPD). Among the 66 pediatric patients who underwent cholangiography, 62 patients had PBM, including 46 cases of type I, 14 cases of type III, and 2 case of type IV (Table 2).

**Table 2:** ERCP interventions.

Events	Values
Failed cannulating	2
Endoscopic sphincterotomy	65
Bile duct stones	61
Methods of stone extraction	
Sphincterotome	43
Stone retrieval basket	15
Stone retrieval balloon	1
Methods of post-ERCP drainage	
ENBD	64
ERPD	1
PBM	
Type 1	46
Type 3	14
Type 4	2

ERCP, endoscopic retrograde cholangiopancreatography; ENBD, endoscopic nasobiliary drainage; ERPD, endoscopic retrograde pancreatic drainage; PBM, pancreaticobiliary maljunction.

### Post-ERCP outcomes

Among the 65 pediatric patients who completed treatment, all except one, who underwent emergency surgery due to unclear relief of postoperative biliary obstruction, achieved good results. In patients with preoperative pancreatitis, postoperative amylase significantly decreased along with noticeable symptom improvement. Pediatric patients with biliary obstruction and concomitant cholangitis showed a significant decrease in liver function index compared to preoperative values (Table 3). The post-ERCP complication rate was 4.41% (3/68), including 1 case of PEP and 2 cases of infection. All patients with post-ERCP complications experienced symptom relief after conservative treatment.

**Table 3:** Comparisons of experimental parameters of patients before and post ERCP.

Events	Before ERCP	Post ERCP	*P*-value
Patients with pancreatitis			
Amylase (U/L)	475.8 ± 460.0	164.5 ± 224.9	<0.001
WBC (1 × 10^9^)	9.9 ± 4.0	9.4 ± 5.2	0.680
Patients with biliary obstruction and cholecystitis			
ALT (U/L)	203.4 ± 133.8	77.3 ± 58.4	<0.001
AST (U/L)	178.2 ± 121.9	92.4 ± 71.1	0.012
γ-GT (U/L)	598.5 ± 466.3	260.0 ± 205.0	0.001
BA (μmol/L)	57.2 ± 70.0	19.23 ± 48.9	0.051
TB (μmol/L)	68.9 ± 60.1	35.5 ± 50.5	0.020
DB (μmol/L)	43.5 ± 48.3	14.6 ± 28.1	0.021
WBC (1 × 10^9^)	14.9 ± 26.1	16.0 ± 25.7	0.883

ERCP, endoscopic retrograde cholangiopancreatography; WBC, white blood cells; ALT, alanine aminotransferase; AST, aspartate aminotransferase; γ-GT, γ-glutamyl transpeptidase; BA, bile acid; TB, total bilirubin; CB, conjugated bilirubin.

### Surgical overview

All 68 pediatric patients underwent definitive surgery. In the 65 patients who completed ERCP treatment, the median interval between surgery and ERCP was 11 days. For children who underwent definitive surgery following ERCP, we all performed cholangiography during the radical surgeries for potential unnoticed stone remnants and fully capturing the complexity of certain PBM cases. The surgical procedure involved standard resection of choledochal cyst followed by hepaticojejunostomy. Among the 68 patients, 49 patients underwent laparoscopic surgery, while 19 patients underwent open surgery. Two patients were converted to open surgery due to severe adhesions found during the procedure. The average duration of surgery was 4.50 ± 3.22 h, with an average blood loss of 20.97 ± 63.16 ml. Blood transfusion was administered to 2 patients intraoperatively. Postoperatively, 7 patients experienced complications, including 3 cases of intra-abdominal infection, 1 case of wound infection, 2 cases of postoperative pancreatitis, and 1 case of chylous fistula. All 7 patients recovered after conservative treatment. The average length of hospital stay postoperatively was 10.5 ± 4.5 days. There were no instances of reoperation or readmission after discharge (Table 4).

**Table 4:** Surgical overview.

Events	Values
Median interval between surgery and ERCP (days)	11
Laparoscopic surgery	49
Conversion to open surgery	2
Duration of surgery (hrs)	4.5 ± 3.2
Blood loss (ml)	21.0 ± 63.2
Blood transfusion	2
Post operation complication	
Intra-abdominal infection	3
Pancreatitis	2
Wound infection	1
Chylous fistula	1
Hospital stay (days)	10.5 ± 4.5
Mortality	0
Re-admission	0
Re-operation	0

ERCP, endoscopic retrograde cholangiopancreatography.

### Timing selection for definitive surgery

To explore the timing selection for definitive surgery following ERCP, we divided the 65 pediatric patients who completed ERCP therapy into early surgery group (≤2 weeks) and late surgery group (>2 weeks). Upon comparison, there were no significant differences between the two groups in terms of surgical duration, rate of minimally invasive surgery, rate of conversion to open surgery, intraoperative bleeding volume, intraoperative blood transfusion, rate of postoperative complications, and average length of hospital stay (Table 5). Therefore, it is inferred that ERCP can be performed early for the treatment of definitive surgery while alleviating preoperative complications of the bile duct.

**Table 5:** Comparisons of parameters between early and late-surgery group.

Events	Early surgery group (*n* = 42)	Late surgery group (*n* = 23)	*P* value
Age (months)	48.0 ± 31.2	48.6 ± 31.1	0.935
Weight (kg)	15.4 ± 6.1	15.2 ± 5.7	0.904
Laparoscopic surgery	32	17	0.838
Conversion to open surgery	2	0	0.537
Duration of surgery (hours)	4.1 ± 0.8	5.3 ± 5.4	0.296
Blood loss (ml)	28.1 ± 79.6	9.0 ± 9.8	0.134
Blood transfusion	2	0	0.542
Post operation complications	5	1	0.411
Hospital stay (days)	10.3 ± 5.3	10.6 ± 2.9	0.819
Mortality	0	0	
Re-admission	0	0	
Re-operation	0	0	

## Discussion

Children with choledochal cyst may experience various complications such as pancreatitis, cholangitis, bile duct perforation, and malignant transformation. These types of choledochal cyst are also known as complex choledochal cyst. The occurrence of complications can affect the prognosis of later treatment. However, standardized approaches for managing complex choledochal cyst have not yet been established. Additionally, the timing of surgery following complication treatment is also an area worthy of exploration. The existing ERCP guidelines are still incomplete with regard to pediatric patients (13). In some pediatric ERCP guidelines, the use of ERCP as a diagnostic tool alone is not recommended (14). Therefore, in this study, all ERCP procedures were therapeutic. In this study, we focused on the two most common complications, pancreatitis and biliary obstruction combined with biliary infection, to investigate the safety and effectiveness of ERCP in treating cyst-related complications. Although children with choledochal cysts complicated by simple pancreatitis can recover with conservative treatment, the waiting time for definitive surgery after pancreatitis is relatively long, and there is a risk of recurrent pancreatitis.

Due to the presence of PBM, pancreatic juice is prone to reflux, leading to the formation of protein plugs in the bile ducts. Protein plugs formed within the common channel ultimately result in the occurrence of pancreatitis (15). According to various reports, the incidence of pancreatitis can range from 5% to nearly 70% (16, 17). Unlike adults with pancreatitis, children with choledochal cyst rarely develop severe pancreatitis. They generally respond well to conservative treatment (18). Although there is no unified guideline for the indications of ERCP in children, it is gradually playing an important role in the diagnosis and treatment of various pediatric pancreatitis cases. ERCP holds high diagnostic and therapeutic value for potential PBM and other pancreatic duct abnormalities. In this study, apart from one case with unsatisfied post-ERCP outcome, the clinical symptoms and elevated amylase levels of the other children with cyst-related pancreatitis were significantly relieved. Additionally, ERCP has significant advantages in managing protein plugs within the common channel, shortening the course of pancreatitis, and laying the foundation for the treatment of the common channel during definitive surgery.

Among the infectious complications of choledochal cyst, cholangitis is common (19), especially in adults with choledochal cyst (20). The exact cause of cholangitis remains unclear, with reports indicating a higher incidence in type 4a choledochal cyst (20). Some patients may respond well to broad-spectrum antibiotics, but there are still some who require timely drainage to relieve obstruction and prevent the adverse consequences of acute suppurative cholangitis. Currently, there are various methods for biliary drainage, including percutaneous drainage or endoscopic drainage (21–23). With the development of pediatric ERCP, its safety and effectiveness have led to more centers opting for ERCP to relieve biliary obstruction. There are no unified guidelines for the diagnosis and treatment of biliary obstruction complicated by cholangitis in pediatric ERCP. Whether ERCP is superior to conservative treatment in terms of treatment success for children with concurrent cholangitis needs further clarification through clinical trials. In this study, all 27 children with choledochal cyst complicated by biliary obstruction received effective treatment, and their symptoms and liver function index showed significant improvement compared to before treatment.

In this study, we did not include patients with type 2 PBM. Patients with type 2 PBM do not have obvious dilation of the bile ducts, and the proportion of symptom occurrence is relatively low. Symptoms also appear later in these patients, so a significant number of type 2 PBM patients often develop the disease in adulthood (24). There is still controversy regarding the treatment of these patients. Whether incidentally discovered PBM requires definitive surgery remains inconclusive. Some researchers believe that surgery is necessary for these patients to avoid symptom occurrence and potential cancer risks. Conversely, others propose that asymptomatic type 2 PBM patients only require observation and follow-up. Additionally, there is ongoing exploration into whether ERCP can avert further surgery in symptomatic type 2 PBM patients (25). The short-term results indicate that ERCP cannot completely prevent symptom recurrence and subsequent definitive surgery (26).

During the inflammatory phase, severe local adhesions can pose challenges to surgery. There is no unified conclusion on how long after inflammation control surgery should be performed. Previous reports suggest that performing surgery 6–8 weeks after inflammation control may be appropriate (6). ERCP can effectively alleviate the symptoms of bile duct cyst, so we speculated whether ERCP could shorten the waiting time for surgery. In recent years, our center generally schedules definitive surgery 1–2 weeks after ERCP. Therefore, we compared the short-term prognosis of patients undergoing surgery at different times, using 2 weeks after ERCP as the dividing point. The results indicate that there is no significant difference in the short-term prognosis between the two groups of patients. Therefore, early definitive surgery after ERCP is safe and feasible. Nevertheless, it is important to highlight that there were 2 cases requiring conversion to open surgery in the early surgery group, and the average blood loss along with the number of transfusion cases were higher compared to those in the late surgery group. While this difference did not reach statistical significance, it implies that the early stage of inflammatory recovery might present challenges during surgery.

Pediatric ERCP can have complications such as PEP, perforation, bleeding, etc., and the occurrence of complications can prolong the hospital stay of children and delay curative surgery. PEP is the most common complication, with an overall incidence rate of approximately 5%–20% (27). PD injection, cannulation and EST, anatomic factors such as pancreas divisum, and patient related factors such as a prior history of PEP and genetic factors may be high-risk factors for PEP (28, 29). In this study, one case of children with biliary obstruction developed PEP, showing elevated amylase levels and worsened abdominal pain after ERCP. This child recovered after conservative treatment. The overall incidence rate of PEP was 1.50% (1/68). Excluding children with pancreatitis as the presenting symptom, the incidence rate of PEP was 3.70% (1/27), which is considerable with other literature reports. According to our center's experience, unlike adults, the incidence of severe post-ERCP pancreatitis in children is lower, and there is still no consensus on preventive measures for postoperative pancreatitis. Further research is needed on the use of preventive drugs such as nonsteroidal anti-inflammatory drugs. In this study, we routinely performed EST on the children. Previous studies have suggested that EST is associated with PEP (29), and as a result, the use of EST in pediatric ERCP has not reached a consensus (30). Additionally, there is a lack of long-term follow-up data to assess the long-term effects of EST on children. Some scholars believe that EST can be considered a standard procedure in children with biliary obstruction and cholangitis (31). In our study, among the 41 children with preoperative pancreatitis, 36 cases had elevated γ-GT and abnormal liver function. We have reason to believe that these children also had biliary obstruction, and the use of EST did not significantly increase the incidence of short-term complications. Future studies with large sample sizes and prospective designs are needed to determine the indications for EST.

This study still has some limitations. Firstly, it is a retrospective study with a relatively small sample size, leading to potential bias in case selection and clinical data collection. Secondly, curative surgeries in this study were performed by multiple surgeons, resulting in certain differences in the timing and methods of surgery following ERCP. Thirdly, based on the data from this study, early surgery after ERCP does not affect the short-term prognosis of pediatric patients. However, whether it affects the occurrence of long-term complications such as intrahepatic bile duct stones, anastomotic strictures, etc., still requires further investigation. Additionally, although we believe that the use of ERCP can provide early definitive surgery for some children with complex choledochal cysts, we did not select children with complex choledochal cysts who had not undergone ERCP treatment as a control group in this study. In the future, we need to design prospective studies and establish multiple control groups to validate our conclusions. Meantime, We only discussed the two most common complications in this study. There is limited research on the treatment of other rare complications with ERCP. Spontaneous bile duct perforation is a rare but serious complication that often requires emergency surgical treatment with cholecystostomy. Portal hypertension is also one of the severe complications of choledochal cyst, and surgery is the only curative method. However, there are reports suggesting that ERCP may serve as one of the treatment options for alleviating symptoms in patients before surgery (32). Finally, the use of ERCP involves several high-cost consumables, which, while providing clinical benefits to patients, also increases their financial burden to some extent. However, the study did not compare the total hospitalization costs and duration.

## Conclusions

In summary, ERCP can safely and effectively alleviate preoperative complications in pediatric patients with choledochal cyst. Early definitive surgery following ERCP does not increase intraoperative risks or postoperative complication rates in pediatric patients. However, further research is needed to investigate the long-term effects of ERCP on pediatric patients with complex choledochal cyst. Additionally, indications for ERCP in pediatric patients with choledochal cyst still require clarification through large-sample prospective studies.

## Data Availability

The raw data supporting the conclusions of this article will be made available by the authors, without undue reservation.
